# Combination therapy with rituximab and cyclophosphamide in the treatment of anti-neutrophil cytoplasmic antibodies (ANCA) positive pulmonary hemorrhage: case report

**DOI:** 10.1186/1546-0096-9-33

**Published:** 2011-10-27

**Authors:** Emily M Baird, Thomas JA Lehman, Stefan Worgall

**Affiliations:** 1 Hospital for Special Surgery, New York, NY and Weil Medical Center, New York, NY, USA; 2 Hospital for Special Surgery, New York, NY and Cornell University, New York, NY, USA; 3 Cornell University, New York, NY, USA

## Abstract

Anti-neutrophil cytoplasmic antibody (ANCA)-associated vasculitis (AAV) with pulmonary hemorrhage is rare in childhood. Standard treatment includes corticosteroids and cyclophosphamide (CYC), which is associated with a high level of toxicity. We report a white female with ANCA positive pulmonary hemorrhage who was treated with cyclophosphamide (CYC) and rituximab (RTX) combination therapy.

## Background

AAV with pulmonary hemorrhage is rare in childhood. Untreated AAV with pulmonary hemorrhage is associated with a 2 year mortality rate in excess of 90% [1]. Standard treatment with a corticosteroid may be associated with medication related complications. We present a case of recurrent pulmonary hemorrhage associated with anti-neutrophil antibody (ANCA)-associated vasculitis (AAV) in childhood. Treatment with cyclophosphamide (CYC) and rituximab (RTX) produced a prompt and sustained remission, and she was able to discontinue steroid therapy.

## Case Presentation

BH, a white female, presented at 7 years of age with fever, cough and respiratory distress following Streptococcal pharyngitis. Her chest radiograph revealed bilateral patchy infiltrates. She was hospitalized with hypoxemia and hypercapnea and required intubation and mechanical ventilation for 14 days. Her endotracheal tube secretions were noted to be bloody and her hemoglobin fell from 10.6 to 8.5 g/dl within 24 hours following admission. The Indices of Coagulation were normal and she was negative for antibodies to anti-nuclear antibody (ANA) and glomerular basement membrane; in addition, protease 3 and myloperoxidase were both negative. There was no hematuria, and C-reactive protein (CRP) was elevated at 12.28 mg/dL, 8.66 mg/dL, and 10.11 mg/dL. A tracheal culture grew Pseudomonas, and chest radiographs showed diffuse airspace opacities with a small right pleural effusion. She was treated with surfactant and dexamethasone, as well as epinephrine and helium in the inspired gas and 17 mg of parenteral methylprednisolone every 6 hours for 14 days. Following a blood transfusion, she slowly stabilized with supportive care and was discharged after eighteen days. Pulmonary hemorrhage was suspected, but a bronchoscopy was refused.

One month after discharge, she complained of rhinorrhea, congestion, cough, stomach pain, and vomiting.

**Figure 1:**
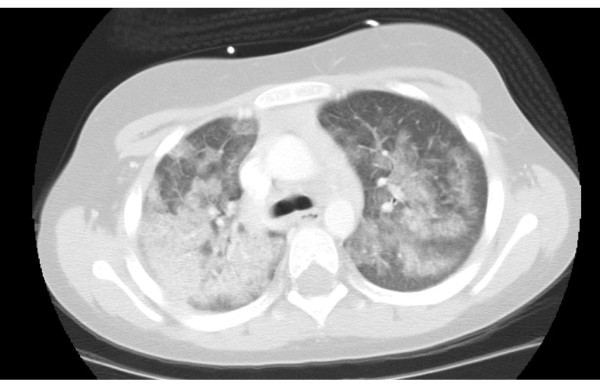
**CT scan at the carina level of the lung showing hemorrhage during the second admission**.

Three months later, she again had Streptococcal pharyngitis. She underwent an elective bronchoscopy to assess for residual pulmonary hemorrhage. The bronchoscopy showed no active bleeding, though subsequently she had an acute PH episode during the bronchoscopy and again required mechanical ventilation. There was no hematuria. Her CRP was 20.40 mg/dL, and she had a positive Epstein-Barr varius titer of 1:160. Her chest computed tomography scan revealed bilateral infiltrates (Figure 1). She was treated with dexamethasone and vasopressors. She again required a blood transfusion. Her symptoms improved, and she was discharged on 1 mg/kg daily of prednisolone after 8 days.

She was treated for 2 months with prednisolone 30 mg daily and subsequently tapered to 7.5 mg every other day. Chest radiographs continued to show bilateral patchy infiltrates, and she had periodic hemoptysis. The reticulocyte count peaked at 5.96 with a hemoglobin of 13.6, 15 months after discharge. Her prednisone dose was increased to 15 mg daily in response to continued bilateral infliltrates on radiograph and reticulocytosis.

**Figure 2:**
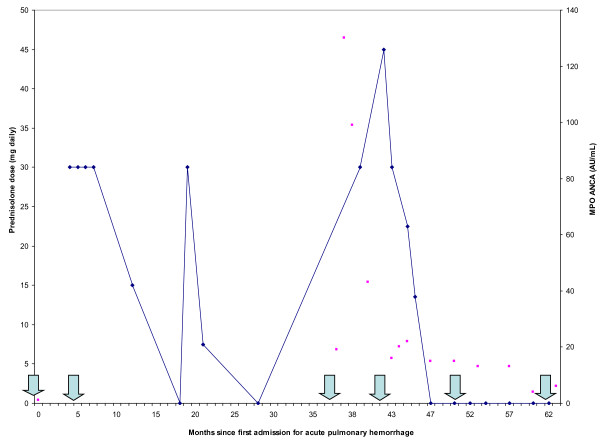
**Steroid dose and myloperoxidase (MPO) IgG ANCA antibodies over time**. The blue line represents prednisolone dose in mg daily, while the scatter plot shows MPO IgG ANCA antibodies in AU/mL. Month 0 is defined as the first admission for pulmonary hemorrhage. The first three arrows indicate the hospitalizations for acute pulmonary hemorrhage, and the last three arrows represent the infusions with CYC and RTX.

Almost 3 years later, at 10 years of age, a chest radiograph showed worsening diffuse airspace opacities, pulmonary interstitial emphysema, and a pneumomediastinum following Streptococcal pharyngitis. She again required admission and mechanical ventilation for pulmonary hemorrhage and respiratory failure. During this admission she tested positive for ANCA Myloperoxidase antibodies by indirect fluorescent antibody assay at 130 AU/mL and 99 AU/mL (Figure 2), with persistently negative ANA and anti glomerular basement membrane antibody. There was no hematuria. The patient had an elevated CRP of 11.75 mg/dL during admission. She was treated with vasopressors and 25 mg of perenteral methylprednisolone every 6 hours. She was intubated for 12 days. Her symptoms improved, and she was discharged after nineteen days on prednisolone of 1 mg/kg daily.

Because she had recurrent episodes of life threatening pulmonary hemorrhage despite 3 years of chronic steroid administration, we elected to treat her aggressively for AAV at 10 years of age. At the time of the first infusion, she was being treated with 45 mg daily of prednisolone. After appropriate discussion of the risks and benefits, consent was obtained and she was hospitalized for CYC and RTX treatment (750 mg/m^2 ^of CYC and 600 mg/m^2 ^of RTX with a maximum of 1000 mg). Following this therapy she was discharged on prednisolone of 30 mg daily which was tapered off gradually over five months. Six and eighteen months after the first infusion, she was retreated with the same dose of CYC and RTX. She tolerated the treatment well. There were no recorded infections or leucopenia. The patient's CRP and reticulocyte count have normalized; the most recent CRP was 0.53 mg/dL. There have been no further episodes of pulmonary hemorrhage, and she remains well without corticosteroid therapy two years following her last infusion.

## Discussion

Diagnosis of AAV was made according to definitions of the Chapel Hill Consensus Conference. The persistently elevated reticulocyte count prior to CYC/RTX therapy suggested that our patient's bleeding was a chronic process. Conventional treatment for this chronic condition includes 3 years of CYC and corticosteroids [2,3].

RTX has recently been investigated as an alternative treatment for AAV [4-8], but two recent studies of RTX in AAV found no clear superiority to CYC [7,9]. The combination of CYC and RTX has been used in an adult with pulmonary hemorrhage secondary to SLE [10]., and we have used it extensively in childhood rheumatic diseases [11-13]. We therefore hypothesized that it might be helpful in our patient with AAV-associated pulmonary hemorrhage. This treatment was given to achieve remission. Alternative therapies of uncertain benefit in this setting might include plasma exchange [14,15], as well as azathioprine or mycophenolate mofetil [16].

## Conclusions

Our patient's remission suggests that combination therapy with CYC and RTX may be effective in this condition. Further studies of the efficacy of this therapy in the treatment of AAV are needed.

## Consent

A waiver of informed consent and waiver of Health Insurance Portability and Accountability Act authorization have been obtained through the local Institutional Review Board.

## Abbreviations

ANCA: anti-neutrophil cytoplasmic antibody; AAV: anti-neutrophil cytoplasmic antibody (ANCA)-associated vasculitis; CYC: cyclophosphamide; RTX: rituximab; ANA: anti-nuclear antibody; CRP: C-reactive protein; m^2^: square meter; g: gram; mg: miligram; dL: deciliter; PH: pulmonary hemorrhage; kg: kilogram; SLE: systemic lupus erythematosus; MPO: myeloperoxidase; IgG: Immunoglobulin G.

## Competing interests

The authors declare that they have no competing interests.

## Authors' contributions

EB wrote the initial manuscript draft. SW assisted in the acquisition of data. All authors reviewed and revised drafts. TL was involved in the interpretation of data and gave approval for the final version to be published. All authors read and approved the final manuscript.
